# Critical thinking dispositions among newly graduated nurses

**DOI:** 10.1111/j.1365-2648.2010.05282.x

**Published:** 2010-10

**Authors:** Sigrid Wangensteen, Inger S Johansson, Monica E Björkström, Gun Nordström

**Affiliations:** 1 Sigrid Wangensteen MSN RN Doctoral Student Department of Nursing, Karlstad UniversitySweden, and Research Fellow Gjøvik University CollegeNorway; 2 Inger S. Johansson PhD RN Associate Professor Department of Nursing, Karlstad UniversitySweden, and Professor Gjøvik University CollegeNorway; 3 Monica E Björkström PhD RN RNT Senior Lecturer Department of Nursing, Karlstad UniversitySweden; 4 Gun Nordström RN PhD Professor Department of Nursing, Karlstad UniversitySweden

**Keywords:** California Critical Thinking Disposition Inventory, critical thinking, newly graduated nurses, Norway, nurse education

## Abstract

wangensteen s., johansson i.s., björkström m.e. & nordström g. (2010) Critical thinking dispositions among newly graduated nurses. *Journal of Advanced Nursing***66**(10), 2170–2181.

**Aim:**

The aim of the study was to describe critical thinking dispositions among newly graduated nurses in Norway, and to study whether background data had any impact on critical thinking dispositions.

**Background:**

Competence in critical thinking is one of the expectations of nursing education. Critical thinkers are described as well-informed, inquisitive, open-minded and orderly in complex matters. Critical thinking competence has thus been designated as an outcome for judging the quality of nursing education programmes and for the development of clinical judgement. The ability to think critically is also described as reducing the research–practice gap and fostering evidence-based nursing.

**Methods:**

A cross-sectional descriptive study was performed. The data were collected between October 2006 and April 2007 using the California Critical Thinking Disposition Inventory. The response rate was 33% (*n*= 618). Pearson’s chi-square tests were used to analyse the data.

**Results:**

Nearly 80% of the respondents reported a positive disposition towards critical thinking. The highest mean score was on the Inquisitiveness subscale and the lowest on the Truth-seeking subscale. A statistically significant higher proportion of nurses with high critical thinking scores were found among those older than 30 years, those with university education prior to nursing education, and those working in community health care.

**Conclusion:**

Nurse leaders and nurse teachers should encourage and nurture critical thinking among newly graduated nurses and nursing students. The low Truth-seeking scores found may be a result of traditional teaching strategies in nursing education and might indicate a need for more student-active learning models.

**What is already known about this topic**

- Critical thinking competence has been designated as an outcome for judging the quality of nursing education programmes and for the development of clinical judgement.
- The ability to think critically is also described as reducing the research–practice gap and fostering evidence-based nursing.
- The California Critical Thinking Disposition Inventory has been used in educational settings and mostly in North America.

**What this paper adds**

- Participants had a positive inclination towards critical thinking, i.e. they were inquisitive, well-informed and orderly in complex matters.
- They scored highest on the Inquisitiveness subscale, indicating that they had intellectual curiosity and a desire for learning.
- They scored lowest on the Truth-seeking subscale, indicating a weak ability to seek new information and a risk of practising based on how things have always been done.

**Implications for practice and/or policy**

- Nurse leaders should encourage and nurture intellectual curiosity and a desire for learning among newly graduated nurses.
- Nurse teachers should encourage critical thinking among nursing students.
- Nurse educators should adopt active learning models and be aware of the relationship between student-active learning models and critical thinking.

## Introduction

Graduate nurses must be critical thinkers with the ability to manage complex situations (Worrell & Profetto-McGrath 2007), and it is expected that nursing education will allow students develop critical thinking dispositions (Daly 1998). Nurse educators are therefore encouraged to evaluate courses and teaching strategies to ascertain whether critical thinking is reflected in their curricula (Girot 2000, Profetto-McGrath 2003). Critical thinking competence has thus been designated as an outcome for judging the quality of nursing programmes (Facione *et al.* 1994, Maynard 1996, Leppa 1997, Thorpe & Loo 2003) and for the development of clinical judgement (Facione & Facione 1997, Smith-Blair & Neighbors 2000, Stone *et al.* 2001). The ability to think critically is also described as reducing the research–practice gap (Seymour *et al.* 2003) and fostering evidence-based nursing (Profetto-McGrath 2005).

## Background

Critical thinking was defined in a Delphi report as a process of purposeful, self-regulatory judgment which results in interpretation, analysis, evaluation and inference (Facione 1990). The report gives this description of an ideal critical thinker:

The ideal critical thinker is habitually inquisitive, well-informed, trustful of reason, open-minded in evaluation, honest in facing personal biases, prudent in making judgements, willing to reconsider, clear about issues, orderly in complex matters, diligent in seeking relevant information, reasonable in the selection of criteria, focused in inquiry and persistent in seeking results which are as precise as the subject and the circumstances of inquiry permit. (Facione 1990, p. 2)

Although there seems to be agreement with respect to the definition of the ideal critical thinker, there are still questions about how to measure critical thinking (Videbeck 1997, Banning 2006). The two most-used instruments are the Watson and Glaser Critical Thinking Appraisal (WGCTA) from 1964 and the California Critical Thinking Disposition Inventory (CCTDI) from 1992 (Banning 2006). The WGCTA is the one most used in nursing (Videbeck 1997, Banning 2006), although it is not specific to nursing (Sedlak 1997). It consists of 80 questions divided into five subscales: inference, recognition of assumptions, deduction, interpretation and evaluation of arguments (Girot 2000). The WGCTA is reported to assess general reasoning skills rather than the discipline-specific thinking learned in a nursing programme (Walsh & Seldomridge 2006).

The CCTDI is designed to measure seven aspects of critical thinking: Truth-seeking, Open-mindedness, Analyticity, Systematicity, Self-confidence, Inquisitiveness and Maturity (Facione 1990). The cross-disciplinary conceptual definition in the Delphi report seems suitable for nursing research (Smith-Blair & Neighbors 2000), although the use of the CCTDI in nursing is limited to date. Stone *et al.* (2001), who investigated nursing students in the final part of their nursing education, reported that nearly all respondents considered the dispositions measured by the CCTDI to be either ‘essential’ or ‘absolutely essential’ for nurses.

We performed a literature review and identified seven empirical studies including graduated nurses and critical thinking, one qualitative and six quantitative studies. Duchscher (2003) reported from her qualitative study that fostering critical thinking is an approach to nursing practice in undergraduate programmes. Three of the quantitative studies used the WGCTA (Maynard 1996, Girot 2000, Hoffman & Elwin 2004). Hoffman and Elwin (2004) found that newly graduated nurses who had high critical thinking scores seemed to be more hesitant in decision-making, while Girot (2000) found no correlation between critical thinking and decision-making. Further, Maynard (1996) reported no relationship between critical thinking ability and professional competence. The remaining three quantitative studies included graduate nurses and used the CCTDI. In one of these (Kawashima & Petrini 2004), statistically significant higher critical thinking dispositions were reported among nursing students compared with experienced nurses with respect to total CCTDI score, and to the Open-mindedness and Inquisitiveness subscales. In addition, Profetto-McGrath *et al.* (2003) reported that nurses who have attributes consistent with the ideal critical thinker were more likely to use research findings in their nursing practice. Although Smith-Blair and Neighbors (2000) recommended further testing of the CCTDI, they also reported that the instrument holds promise with respect to assisting nurse educators in developing induction programmes.

May *et al.* (1999) recommended studying critical thinking in a setting where newly graduated nurses have ‘real world’ experience as nurses, and Redding (2001) pointed out that critical thinking dispositions may not become readily apparent until after graduation. Of the seven studies mentioned above only one (Girot 2000), using the WGCTA, was carried out in a European context. The CCTDI has been used in a European study including nursing students (Ozturk *et al.* 2008), but has to our knowledge not been used in studies with graduated nurses.

## The study

### Aim

The aim of this study was to describe critical thinking dispositions by means of the CCTDI among newly graduated nurses in Norway, and to study whether background data had any impact on critical thinking dispositions.

### Design

A cross-sectional descriptive study was performed, and two questionnaires were used: a study-specific questionnaire for background data and the CCTDI.

### Participants

The study population were newly graduated nurses from 27 university colleges in Norway in June 2006 (*N*= 2675), and the inclusion criterion was to be working as a nurse. To check the procedure for mailing and response rate, a pilot study was performed. This revealed that newly graduated nurses move and consequently change their addresses, resulting in a large volume of return-to-sender mail. To reduce the return-to-sender mail, all addresses were compared with those in the membership register of the Norwegian Nurses’ Association.

A power analysis was done and this showed that, to obtain a statistical power of 79·6% with statistical significance (alpha) set at 0·050 (two-tailed), 730 respondents were needed. Based on these numbers and a response rate of about 50%, we calculated that 1500 questionnaires would need to be sent out. From the 27 university colleges in Norway, those in this study were chosen by drawing lots, and the number of graduated nurses from each college was noted. This process continued until the recommended number of potential respondents was obtained.

**Figure 1:**
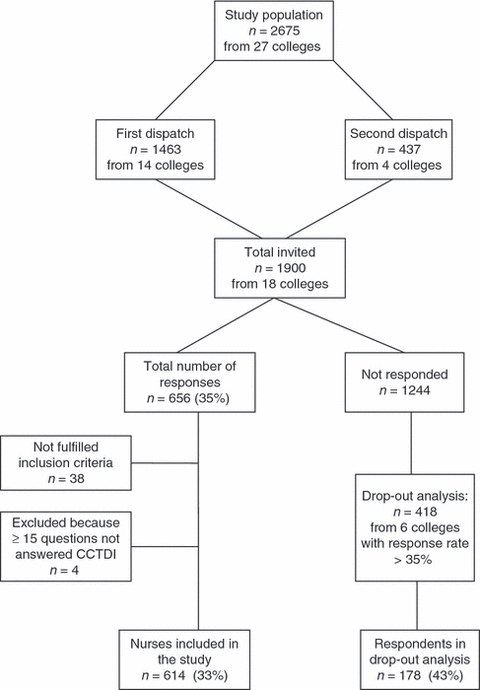
Overview of study population, sample and response rate, including respondents in the drop-analysis.

Figure 1 shows an overview of the study population, sample and respondents in a drop-out analysis (see below). The first mailing (October 2006) included 1463 nurses graduated from 14 university colleges. As a result of a low response rate in this mailing, four additional university colleges were included by drawing further lots (*n*= 437) (November 2006). In total, all nurses (*n*= 1900) from 18 university colleges (14 + 4) were asked to participate and two reminders were sent. Of the total of 656 respondents, 38 were excluded because they did not fulfil the inclusion criteria (did not work as nurses). Another four were excluded because they had left 15 or more questions unanswered on the CCTDI. In total, 614 nurses (33%) were included in the study.

#### Drop-out analysis

As a result of the low response rate (33%), a drop-out analysis was performed. The six colleges with a response rate >35% were included in this analysis. All nurses who had not responded after two reminders from these six colleges (*n*= 418) received a questionnaire including questions about age, gender, whether they had been educated at university college level and their healthcare experience prior to nursing education. A total of 178 responded (43%) (Figure 1). In addition, data about gender and age of all graduated nurses in Norway in June 2006 (*N*= 2675) were obtained from the Norwegian Health Personnel Register.

### Data collection

Data collection was carried out between October 2006 and April 2007 by means of a study-specific questionnaire and CCTDI. The study-specific questionnaire contained questions about gender, age, work area (i.e. general or mental health hospitals and community health care), education at university college level and healthcare experience (in either case prior to nursing education).

The CCTDI consists of 75 statements in seven subscales with 9–12 items in each of the subscales (Facione *et al.* 2001). An overview of the CCTDI subscales, components and examples of statements is shown in Table 1. The items in the seven subscales are interspersed throughout the instrument (Facione *et al.* 2001).

**Table 1:** California Critical Thinking Dispositions Inventory subscales – components and examples of statements

Subscale	Component*	Example of statement	Relevance to nursing†
Truth-seeking	Eager to seek the truth, courageous about asking questions, honest and objective about pursuing inquiry even if the findings do not support one’s interest or one’s preconceived opinions	It is never easy to decide between competing points of view	A truth-seeking nurse continually re-evaluates new information and evidence
Open-mindedness	Open-minded and tolerant of divergent views with sensitivity to the possibility of one’s bias. Respect the rights of others to hold different opinions	It concerns me that I might have biases of which I’m not aware	Dispositional intolerance of divergent views might preclude effective nursing interventions
Analyticity	Alert to potentially problematic situations, anticipating possible result or consequences, and prizing the application of reason and the use of evidence even if the problem at hand turns out to be challenging and difficult	It bothers me when people rely on weak arguments to defend good ideas	Being analytical allows the nurse to connect clinical observations with her or his theoretical knowledge base, and to anticipate events likely to threaten the safety or limit health potential of a given individual
Systematicity	Disposition towards organized, orderly, focused, and diligent inquiry	I always focus on the question before I attempt to answer it	Organized approaches are an indispensable part of clinical practice, and deficit in systematicity might particularly predispose a nurse to the possibility of negligence in practice
Self-confidence	Level of one’s trust one place in one’s own reasoning processes	I take pride in my ability to understand the opinions of others	An appropriate level of CT self-confidence would be desired trajectory in the nursing student and nurse clinician. Nurses who overrate their CT abilities may act with inadequate caution, while those whose CT confidence is lower than actual CT skills might be expected to demonstrate lack of leadership
Inqusitiveness	One’s intellectual curiosity and one’s desire for learning even when the application of the knowledge is not readily apparent	Learn everything you can, you never know when it could be handy	Considering that the knowledge base for competent nursing practice continues to expand, a deficit in inquisitiveness would signal a fundamental limitation of one’s potential to develop expert knowledge and clinical practice ability
Maturity	The CT mature person approaches problems, inquiry, and decision-making with a sense that some problems are necessarily ill-structured. Many times judgments must be based on standards, context and evidence which preclude certainty	The best way to solve problems is to ask someone else for the answer	This disposition has particular implications for ethical decision-making, particularly in time-pressured environments

CT, critical thinking.

* Sources: Facione *et al.* (1995, 2001).

† Source: Facione *et al.* (1994, p. 346–347).

The instrument uses a 6-point Likert scale in which 1 = strongly agree and 6 = strongly disagree. Total scores range between 70 and 420, while subscale scores range from 10 to 60. To calculate subscale scores, raw scores are multiplied by 10 and divided by the number of items in the subscale (Profetto-McGrath 2003). The higher the score, the stronger disposition towards critical thinking. A total score above 350 indicates a strong disposition, while a score between 280 and 350 indicates a positive inclination (i.e. high critical thinking score). Total scores between 210 and 279 fall in the ambivalent range, while scores below 210 indicate strong opposition towards critical thinking (i.e. low critical thinking scores) (Facione *et al.* 2001). Subscale scores above 50 indicate a strong disposition, scores between 40 and 50 a positive inclination (i.e. high subscale scores), scores between 30 and 39 ambivalence, while scores below 30 indicate a strong opposition towards critical thinking (i.e. low subscale scores) (Facione *et al.* 2001).

For this study, the CCTDI was translated into Norwegian using to the following steps: (i) the original English instrument was translated into Norwegian by the researcher, a native Norwegian (SW), (ii) the Norwegian version was translated back into English by a bilingual professional person who had not seen the original English version and (iii) the three versions were then compared. Unclear or incorrect translations were discussed between the researcher and the professional translator until agreement was obtained. Thus, the translation process followed the recommendations provided by the California Academic Press and according White and Elander (1992).

### Ethical considerations

The study was carried out according to the Ethical Guidelines for Nursing Research in the Nordic Countries (Northern Nurses’ Federation 2003). It was approved by the Ethics Committee at the University, and by the Norwegian Social Science Data Services. The Norwegian Nurses’ Association gave permission to access the membership register.

### Data analysis

The spss Version 15·0 for Windows was used for the analyses, and both descriptive and inferential statistics were used. According to the directions for analysing the CCTDI given by Insight Assessment, unanswered questions were given the value 3·5 when fewer than 15 questions were unanswered. When 15 or more questions were unanswered, respondents were excluded from the study (Figure 1). To check if CCTDI total scores and subscale scores were normally distributed, the Kolmogorov-Smirnov test was performed (Field 2005). The CCTDI total score was normally distributed. As a result of the fact that the CCTDI is based on ordinal data (Likert scale) and that the subscale scores were not normally distributed, nonparametric tests were used. To test differences in proportions between groups, i.e. respondents with high vs. low critical thinking scores in relation to background variables measured at a categorical level, Pearson’s chi-square tests were carried out. This test was also used to check differences in proportions between the study sample and the drop-out respondents regarding gender, university college education and working experience in the healthcare sector prior to nursing education (Altman 1991). Age differences between the study sample and respondents in the drop-out analysis were calculated using the Mann–Whitney *U*-test. The level of statistical significance was set at *P*< 0·05.

### Validity and reliability

The reliability of CCTDI has been measured using Cronbach’s alpha in different populations. Alpha values for the total score in two studies including college students were 0·90 (Walsh & Hardy 1999) and 0·91 (Facione *et al.* 2001), and in studies with nursing students 0·76 (May *et al.* 1999) and 0·85 (Ip *et al.* 2000, Ozturk *et al.* 2008). In studies including graduate nurses, the values were 0·87 (Smith-Blair & Neighbors 2000) and lower than 0·80 (Profetto-McGrath *et al.* 2003). Cronbach’s alpha values for the present study are reported in Table 2.

**Table 2:** Critical thinking dispositions (CCTDI total- and subscale scores) for newly graduated nurses in Norway (*n*= 614)

CCTDI scores	Mean	Median	sd	Min.–max.	Cronbach’s alpha
Total score*	300·3	301·0	24·78	228–380	0·83
Subscales					
Truth-seeking†	39·4	39·0	5·85	18–59	0·60
Open-mindedness†	40·9	41·0	5·45	26–58	0·46
Analyticity†	42·9	43·0	4·84	26–55	0·48
Systematicity†	45·5	45·5	6·18	13–59	0·64
CT self-confidence†	41·2	42·0	6·53	19–57	0·72
Inquisitiveness†	48·0	49·0	5·67	28–60	0·60
Maturity†	42·4	43·0	6·02	20–59	0·52

Mean values, median values, standard deviation (sd), minimum and maximum values (min.–max.), and Cronbach’s alpha values are shown.

* Critical thinking total mean scores indicating: strong disposition >350, positive inclination 280–350, ambivalent 210–279, strong opposition <210.

† Critical thinking subscale mean scores indicating strong disposition >50, positive inclination 40–50, ambivalent 30–39, strong opposition <30.

The grounding of the CCTDI in the previously mentioned Delphi study (Facione 1990) supports its validity. Furthermore, Facione and Facione (1997) referred to the work of Giancarlo (1996), who found a statistically significant positive correlation between CCTDI and ego-resilience and also between the CCTDI and openness to experience (Facione & Facione 1997). Further, the CCTDI correlates with measures of personality and academic achievement (Giancarlo & Facione 2001).

## Results

The mean age of the participating nurses was 30·9 years. When comparing the mean age of the study sample (30·9 years; sd 8·67) and respondents in the drop-out analysis (30·2 years; sd 8·23), no statistically significant difference was seen. The mean age of the study population was 30·5 years (sd 8·03). Further, no statistically significant differences were found between the study sample and the respondents in the drop-out analysis with respect to university college education or healthcare experience prior to nursing education concerning background variables (Table 3).

**Table 3:** Comparisons between (a) the study sample (*n*= 614), (b) respondents in the dropout analysis (*n*= 178) and (c) the study population (*N*= 2675) with respect to background data

	(a) *n*= 614, *n* (%)	(b) *n*= 178, *n* (%)	(c) *N*= 2675, *n* (%)	Pearsons χ^2^ test
Gender				
Female	556 (90·6)	153 (86·0)	2415 (90·3)	a/b, a/c, b/c; NS
Male	58 (9·4)	25 (14·0)	260 (9·7)	
University prior to nursing education				
Yes	114 (18·6)	43 (24·2)		NS
No	492 (80·1)	135 (75·8)		
Healthcare experience prior to nursing education				
Yes	369 (60·1)	113 (63·5)		NS
No	242 (39·4)	65 (36·5)		

NS, not significant.

### Critical thinking dispositions

Critical thinking dispositions in the study sample are shown in Table 2. The mean value of the total CCTDI score was 300·3, indicating a positive inclination towards critical thinking. Six of the seven subscale mean scores were above 40, the recommended cut-off score, also indicating a positive inclination. The highest-rated mean score was found on the Inquisitiveness subscale (48·0), characterizing an intellectual curiosity and desire for learning, and the lowest-rated mean score on the Truth-seeking subscale (39·4), indicating ambivalence related to seeking the best knowledge and courage to ask questions.

**Figure 2:**
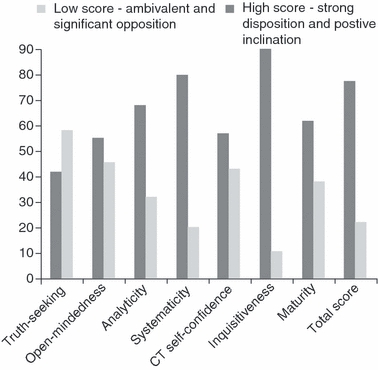
Critical thinking dispositions among newly graduated nurses in Norway (*n*= 614). Per cent of respondents (*y*-axis) with high and low scores for subscales* and total score^†^ (*x*-axis) are shown graphically. *High critical thinking subscale scores i.e.strong disposition >50 and positive inclination 40–50, and low critical thinking subscale scores i.e. ambivalent 30–39 and strong opposition <30. ^†^High critical thinking total scores i.e. strong disposition >350 and positive inclination 280–350 and low critical thinking total scores i.e. ambivalent 210–279 and strong opposition <210.

When dichotomizing total CCTDI scores into high (i.e. strong disposition and positive inclination) and low (i.e. ambivalent and strong opposition towards critical thinking), nearly 80% of the respondents fell into high score group (i.e. 280 or higher), while approximately one-fifth (22%) fell into the low score group (i.e. 279 or lower) (Figure 2). No respondents reported strong opposition towards critical thinking.

A large majority of the participants, 90% and 80% respectively, scored above the recommended cut-off score of 40 on the Inquisitiveness and Systematicity subscales (Figure 2). The corresponding figures for the Analyticity and Maturity subscales were 68% and 62% respectively. The Truth-seeking subscale was the only subscale where the majority of the sample (58%) scored below the recommended cut-off score of 40.

### Critical thinking dispositions and background data

A statistically significant greater proportion of nurses older than 30 years reported high CCTDI total scores compared with those younger than that (Table 4). This was also the case for the CCTDI subscales Truth-seeking, Systematicity and Inquisitiveness. When comparing critical thinking dispositions between males and females a statistically significant greater proportion of the former group reported high values on the Analyticity subscale. Nurses with university education prior to nursing education reported to greater extent high values on the total CCTDI and the Truth-seeking subscale. Further, those working in community health care reported to a greater extent high score on the total CCTDI and the Systematicity subscale.

**Table 4:** Statistically significant differences in proportions (no. presented in per cent) of nurses with high critical thinking scores on the California Critical Thinking Disposition Inventory (CCTDI) total (i.e. ≥280) and subscales scores (i.e. ≥40) related to background data (Pearson’s chi-square test)

	%	χ^2^	*P* value
Age			
>30 years (*n*= 249) vs. ≤30 years (*n*= 361)*			
CCTDI total score	88 vs. 75	14·44	<0·001
Truth-seeking	55 vs. 42	9·44	0·002
Systematicity	88 vs. 80	6·19	0·013
Inquisitiveness	96 vs. 91	5·39	0·020
Gender			
Males (*n*= 58) vs. females (*n*= 556)			
Analyticity	88 vs. 73	6·01	0·014
University education prior to nursing education			
Yes (*n*= 114) vs. no (*n*= 492)†			
CCTDI total score	87 vs. 79	4·07	0·044
Truth-seeking	65 vs. 43	17·36	<0·001
Work area			
Community health care (*n*= 222) vs. hospitals (*n*= 340)‡			
CCTDI total score	86 vs. 77	6·92	0·009
Systematicity	88 vs. 81	5·43	0·020

* Four respondents did not answer the question (*n*= 610).

† Eight respondents did not answer the question (*n*= 606).

‡ Forty-nine respondents (8%) had part-time positions in both hospitals and community health care (not reported here). Three respondents did not answer the question (*n*= 562).

## Discussion

### Study limitations

This study included 614 newly graduated nurses in Norway, representing a response rate of 33%. This response rate led to a drop-out analysis. A total of 178 nurses responded to questions about age, gender and whether or not they had education at university college level and healthcare experience prior to nursing education. This analysis and the information about age and gender for the study population (*N*= 2675) revealed that there were no statistically significant differences between these groups with respect to the background variables. This is an aspect of internal validity, and contributes to strengthening the results. One reason for the low response rate may be the fact that, in addition to the CCTDI, two other questionnaires were sent at the same time (these results will be reported elsewhere). One of these questionnaires was quite extensive. Another possible reason for drop-out may have been lack of interest in the study topic.

In the present study the Cronbach’s alpha value for the total CCTDI was 0·83, indicating a good internal consistency (Burns & Grove 2001). This is in line with the findings of other authors using the CCTDI outside the United States of America (USA) and Canada (Ip *et al.* 2000, Tiwari *et al.* 2003, Ozturk *et al.* 2008). Our alpha values for the subscales varied between 0·46 and 0·72. Low subscale values have also been reported by other authors (Leppa 1997, Ip *et al.* 2000, Tiwari *et al.* 2003, Ozturk *et al.* 2008). Kawashima and Petrini (2004) suggested in their Japanese study that low Cronbach’s alpha values might be as a result of cultural biases. Even though the Norwegian culture is thought to be more like the US culture than the Japanese, cultural bias might also have affected findings in the present study. We recommend more studies using the CCTDI in a European context to test the reliability further in this context. Regarding external validity, cluster sampling was used, as the colleges graduating the nurses included in the study were chosen by drawing lots (Figure 1). Cluster sampling is a random sampling method and reduces sampling error (Burns & Grove 2001).

### Discussion of results

In this study we focused on critical thinking dispositions among newly graduated nurses in Norway and relationships between the background data and critical thinking dispositions. Development of critical thinking dispositions is essential to enable newly graduated nurses to function as professional nurses (Thorpe & Loo 2003), and an ideal critical thinker has been described as inquisitive, well-informed, open-minded, willing to reconsider and orderly in complex matters (Facione 1990). This description might well be a description of the ‘ideal’ nurse.

The respondents in this study reported mean overall CCTDI scores indicating a positive inclination towards critical thinking. When comparing our findings to studies including nursing students, our newly graduated nurses scored lower than US and Canadian nursing students (May *et al.* 1999, Profetto-McGrath 2003), but higher than nursing students from Hong Kong and Australia (Tiwari *et al.* 2003) and Turkey (Ozturk *et al.* 2008). Total CCTDI score in the present study was higher than that reported for nurses (the sample included nurses at different educational levels, and some were assistant nurses) in a Canadian (Profetto-McGrath *et al.* 2003) and Japanese study (Kawashima & Petrini 2004), but lower than those for US nurses (Facione & Facione 1997, Smith-Blair & Neighbors 2000). These findings might reflect the cultural differences mentioned above.

The highest subscale mean score (48·0) was found on the Inquisitiveness subscale. Here nearly 90% of the nurses were positively disposed (i.e. had scores above the cut-off score of 40), which is in line with other studies (May *et al.* 1999, Smith-Blair & Neighbors 2000, Profetto-McGrath 2003). This subscale measures intellectual curiosity and desire for learning. A deficit in this respect would indicate a fundamental limitation of ‘one’s potential to develop expert knowledge and clinical practice ability’ (Facione *et al.* 1994, p. 346), and nurses scoring high on this subscale are motivated to expand their knowledge bases (Smith-Blair & Neighbors 2000). According to Profetto-McGrath (2003), this finding reflects eagerness to obtain knowledge even when it may not have immediate use, a finding that is encouraging and desirable. The disposition towards inquisitiveness among nurses in the present study seems to correspond with findings in a qualitative study including newly graduated nurses, who reported that they looked upon challenges as opportunities for learning (Wangensteen *et al.* 2008). People who have a strong belief in their capabilities tend to approach difficult tasks as challenges to be mastered (Bandura 1997). Further, nurses who are inquisitive, open-minded and systematic are more likely to use research findings in their work, which may contribute to high-quality nursing care (Profetto-McGrath *et al.* 2003).

The lowest mean score was found on the Truth-seeking subscale (39·4). More than half of our respondents scored between 30 and 39 on this subscale, and approximately 5% scored below 30, indicating strong opposition in this respect. Low scores on the Truth-seeking subscale may be seen in nurses who are unwilling to re-evaluate new information, and who base their nursing on ‘how things always have been done’ (Smith-Blair & Neighbors 2000). Other authors have also reported the lowest mean scores for this subscale (May *et al.* 1999, Ip *et al.* 2000, Smith-Blair & Neighbors 2000, Profetto-McGrath *et al.* 2003, Tiwari *et al.* 2003). Further, Walsh and Hardy (1999), who studied students on six academic programmes, reported the lowest mean score (below 40) for the Truth-seeking subscale. This was the case across all six programmes, but the mean score for nursing students was reported to be lowest. As the Truth-seeking subscale targets intellectual honesty (Giancarlo & Facione 2001), i.e. the disposition to be courageous about asking questions and to be honest and objective about pursuing inquiry even when the topics do not support one’s self-interest (Facione *et al.* 1994), these findings are worrying. The low mean score for this subscale has been explained in several studies by questioning whether nursing programmes still have traditional and strictly didactic teaching strategies (May *et al.* 1999, Walsh & Hardy 1999, Profetto-McGrath *et al.* 2003). It would be desirable that newly graduated nurses had higher scores with respect to Truth-seeking, as a higher disposition would indicate a capability to re-evaluate new information and not base practice on how procedures have always been done. Despite low Truth-seeking mean scores, Ozturk *et al.* (2008) reported statistically significant higher scores for nursing students in a problem-based learning (PBL) model (40·1) compared with those following a traditional educational model (35·8). These authors also discussed the emphasis on questioning and information-seeking skills in the PBL model as a possible explanation for this difference. It would therefore be of interest to study this relationship further. There might be a need for a new curriculum in nursing, with learning models based on active student participation and where critical thinking is an important element (Bevis & Watson 2000). Further, May *et al.* (1999) questioned whether the standard score for the Truth-seeking subscale has been established at a higher level than might reasonably be expected.

In our study, nurses older than 30 years to a greater extent reported high values on the total CCTDI, as well as on three of the subscales compared with those younger than that age. Tiwari *et al.* (2003) reported corresponding results for the CCTDI total score for nursing students. Facione and Facione (1997) reported statistically significant correlations between age and several CCTDI subscales (i.e. the older the higher scores), but only with respect to the Truth-seeking subscale was the correlation high enough (*r* = 0·225) to be noteworthy. Walsh and Hardy (1999) reported no statistically significant gender differences with respect to CCTDI total score or subscale scores. In contrast to findings in some other studies that women scored statistically significant higher than men on the Open-mindedness and Maturity subscales (Facione *et al.* 1995, Facione & Facione 1997, Giancarlo & Facione 2001), no such gender differences were found in the present study. However, there were statistically significant more males than females with high scores on the Analyticity subscale, a finding in line with that of Giancarlo and Facione (2001). Despite the gender differences reported, Giancarlo and Facione (2001) claim that males and females are notably similar with respect to critical thinking.

In the present study, approximately two-thirds of nurses with university education prior to nursing education reported high scores on the Truth-seeking subscale compared with less than half of those without such education. Pepa *et al.* (1997), who measured critical thinking by means of the WGCTA, reported that students who had completed 44 college credits prior to nursing education were able to think more critically than those without such education. Comparisons between those with and without university education prior to nursing education have not been found in previous CCTDI studies. Despite the low scores on the Truth-seeking subscale in the present, as well as in others (May *et al.* 1999, Walsh & Hardy 1999, Profetto-McGrath *et al.* 2003), our findings indicate that university education prior to nursing education might have an impact on the Truth-seeking subscale. Sixty per cent of our newly graduated nurses had healthcare experience prior to nursing education. This experience, however, did not seem to contribute to their critical thinking.

A greater proportion of nurses working in community health care reported high scores on the total CCTDI compared with the nurses working in hospitals. One explanation for this might be that those working in community health care were older (mean age 32·7) than those working in hospitals (mean age 29·6). Thirty-six per cent of the newly graduated nurses in the present study worked in community health care, an area where the number of patients needing care at a high professional level is increasing (Kalseth *et al.* 2004). Long-term care nursing is reported to be a complex, demanding and interesting nursing work environment (Leppa 2004), and is also described as being a ‘fast-growing industry’ (Bevis & Watson 2000). Thus, having newly graduated nurses with a positive inclination to be critical thinkers, i.e. inquisitive, well-informed and orderly in complex matters, will be of benefit in community health care.

The present study demonstrated some differences between nurses with high vs. low critical thinking dispositions with regard to background variables as age, gender, university education prior to nursing education and work area. Fero *et al.* (2009), who studied critical thinking ability among newly graduated and experienced nurses, regretted that this kind of information was not available in their study, recommending those variables to be included in further studies.

#### Critical thinking and nursing practice

Critical thinking in nursing is an essential component of professional accountability and quality nursing care (Distler 2007), an outcome expected of all graduate nurses (Pepa *et al.* 1997). Individuals who have developed the disposition for truth-seeking, open-mindedness, analyticity, systematicity, self-confidence, inquisitiveness and maturity are more likely to apply critical thinking in their personal and professional lives (Smith-Blair & Neighbors 2000).

Skills alone do not guarantee success in the workplace, because people must be disposed to use what they have learnt (Facione *et al.* 2000). This statement is in line with Bandura (1997) concept of self-efficacy, described as a belief about how to manage different situations in different contexts. Nurses who are critical thinkers may contribute to changing health care to improve it (Bevis & Watson 2000). Furthermore critical thinking is reported as vital to evidence-based nursing (Profetto-McGrath 2005). Daly (1998) claims that critical thinking from an interdisciplinary point of view would contribute to sound professional relationships and political awareness, a statement in line with the expectation that nurses should identify themselves as partners in an interdisciplinary team (Krøll & Hansen 2000).

## Conclusion

Intellectual curiosity is important, especially in professional areas where the knowledge base is constantly expanding. Thus, it is of utmost importance both for teachers in nursing education and for nurse leaders in clinical practice to nurture this curiosity and desire for learning. Nurse educators are encouraged to use student-active learning models and be aware of the relationship between teaching strategies and critical thinking. Supervision from experienced nurses who are able to nurture intellectual honesty might contribute to increased critical thinking dispositions among newly graduated nurses.

## References

[b1] Altman D (1991). Practical Statistics for Medical Research.

[b2] Bandura A (1997). Self-Efficacy. The Exercise of Control.

[b3] Banning M (2006). Nursing research: perspectives on critical thinking. British Journal of Nursing.

[b4] Bevis EO, Watson J (2000). Toward a Caring Curriculum. A New Pedagogy for Nursing.

[b5] Burns N, Grove SK (2001). The Practice of Nursing Research. Conduct, Critique & Utilization.

[b6] Daly WM (1998). Critical thinking as an outcome of nursing education. What is it? Why is it important to nursing practice?. Journal of Advanced Nursing.

[b7] Distler JW (2007). Critical thinking and clinical competence: results of the implementation of student-centered teaching strategies in an advanced practice nurse curriculum. Nurse Education in Practice.

[b8] Duchscher JEB (2003). Critical thinking: perceptions of newly-graduatedfemale baccalaureate nurses. Journal of Nursing Education.

[b9] Facione PA (1990). Critical Thinking: A Statement of Expert Consensus for Purposes of Educational Assessment and Instruction. Executive Summary “The Delphi Report”.

[b10] Facione NC, Facione PA (1997). Critical Thinking Assessment in Nursing Education Programs: An Aggregate Data Analysis.

[b11] Facione NC, Facione PA, Sanchez CA (1994). Critical Thinking Dispositions as a measure of competent clinical judgment: the development of the critical thinking disposition inventory. Journal of Nursing Education.

[b12] Facione PA, Sanchez CA, Facione NC, Gainen J (1995). The disposition toward critical thinking. The Journal of General Education.

[b13] Facione PA, Facione NC, Giancarlo CA (2000). The disposition toward critical thinking: its character, measurement, and relationship to critical thinking skill. Informal Logic.

[b14] Facione P, Facione N, Giancarlo C (2001). California Critical Thinking Disposition Inventory Test Manual.

[b15] Fero LJ, Witsberger CM, Wesmiller SW, Zullo TG, Hoffman LA (2009). Critical thinking ability of new graduate and experienced nurses. Journal of Advanced Nursing.

[b16] Field A (2005). Discovering Statistics Using SPSS.

[b17] Giancarlo CAF (1996). The Ideal Critical Thinking: Development of an Expert Q-Sort Prototype.

[b18] Giancarlo CA, Facione PA (2001). A look across four years at the disposition toward critical thinking disposition among undergraduate students. The Journal of General Education.

[b19] Girot EA (2000). Graduate nurses: critical thinkers or better decisions makers?. Journal of Advanced Nursing.

[b20] Hoffman K, Elwin C (2004). The relationship between critical thinking and confidence in decision making. Australian Journal of Advanced Nursing.

[b21] Ip WY, Lee DT, Lee IFK, Chau JPC, Wootton YSY, Chang AM (2000). Disposition towards critical thinking: a study of Chinese undergraduate students. Journal of Advanced Nursing.

[b22] Kalseth B, Midttun L, Paulsen B, Nygård L (2004). Utviklingstrekk i kommunehelsetjenesten og spesialisthelsetjenesten – oppgaveutvikling og samspill (Development in Community Health Care and Specialist Health Care – Tasks and Cooperation).

[b23] Kawashima A, Petrini MA (2004). Study of critical thinking skills in nursing students and nurses in Japan. Nurse Education Today.

[b24] Krøll V, Hansen H (2000). The competence of the newly qualified nurse now and in five years – seen from an empirical and professional perspective. Nursing Science and Research in the Nordic Countries.

[b25] Leppa CJ (1997). Standardized measures of critical thinking. Experiences with the California critical thinking tests. Nurse Educator.

[b26] Leppa CJ (2004). The nature of long-term care nursing work. Journal of Gerontological Nursing.

[b27] May BA, Edell V, Butell S, Doughty J, Langford C (1999). Critical thinking and clinical competence: a study of their relationship in BSN seniors. Journal of Nursing Education.

[b28] Maynard CA (1996). Relationship of critical thinking ability to professional nursing competence. Journal of Nursing Education.

[b29] Northern Nurses’ Federation (2003). Ethical Guidelines for Nursing Research in the Nordic Countries.

[b30] Ozturk C, Muslu GK, Dicle A (2008). A comparison of problem-based and traditional education on nursing students’ critical thinking dispositions. Nurse Education Today.

[b31] Pepa CA, Brown JM, Alverson EM (1997). A comparison of critical thinking abilities between accelerated and traditional baccalaureate nursing students. Journal of Nursing Education.

[b32] Profetto-McGrath J (2003). The relationship of critical thinking skills and critical thinking dispositions of baccalaureate nursing students. Journal of Advanced Nursing.

[b33] Profetto-McGrath J (2005). Critical thinking and evidence-based practice. Journal of Professional Nursing.

[b34] Profetto-McGrath J, Hesketh KL, Lang S, Estabrooks CA (2003). A study of critical thinking and research utilization among nurses. Western Journal of Nursing Research.

[b35] Redding DA (2001). Spotlight on: critical thinking disposition as it relates to academic achievement in baccalaureate nursing education. Nurse Educator.

[b36] Sedlak CA (1997). Critical thinking of beginning baccalaureate nursing students during the first clinical nursing course. Journal of Nursing Education.

[b37] Seymour B, Kinn S, Sutherland N (2003). Valuing both critical and creative thinking in clinical practice: narrowing the research-practice gap?. Journal of Advanced Nursing.

[b38] Smith-Blair N, Neighbors M (2000). Use of the critical thinking disposition inventory in critical care orientation. The Journal of Continuing Education in Nursing.

[b39] Stone CA, Davidson LJ, Evans JL, Hansen MA (2001). Validity evidence for using a general critical thinking test to measure nursing students’ critical thinking. Holistic Nursing Practice.

[b40] Thorpe K, Loo R (2003). Critical-thinking types among nursing and management undergraduates. Nurse Education Today.

[b41] Tiwari A, Avery A, Lai P (2003). Critical thinking disposition of Hong Kong Chinese and Australian nursing students. Journal of Advanced Nursing.

[b42] Videbeck SL (1997). Critical thinking: prevailing practice in baccalaureate schools of nursing. Journal of Nursing Education.

[b43] Walsh CA, Hardy RC (1999). Dispositional differences in critical thinking related to gender and academic major. Journal of Nursing Education.

[b44] Walsh CM, Seldomridge LA (2006). Measuring critical thinking: one step forward, one step back. Nurse Educator.

[b45] Wangensteen S, Johansson IS, Nordström G (2008). The first year as a nurse – an experience of growth and development. Journal of Clinical Nursing.

[b46] White M, Elander G (1992). Translation of an instrument. Scandinavian Journal of Caring Sciences.

[b47] Worrell JA, Profetto-McGrath J (2007). Critical thinking as an outcome of context-based learning among post RN students: a literature review. Nurse Education Today.

